# Characterization of Novel Factors Involved in Swimming and Swarming Motility in *Salmonella enterica* Serovar Typhimurium

**DOI:** 10.1371/journal.pone.0135351

**Published:** 2015-08-12

**Authors:** Julia Andrea Deditius, Sebastian Felgner, Imke Spöring, Caroline Kühne, Michael Frahm, Manfred Rohde, Siegfried Weiß, Marc Erhardt

**Affiliations:** 1 Junior Research Group Infection Biology of *Salmonella*, Helmholtz Centre for Infection Research, Inhoffenstraße 7, 38124 Braunschweig, Germany; 2 Department of Molecular Immunology, Helmholtz Centre for Infection Research, Inhoffenstraße 7, 38124 Braunschweig, Germany; 3 Central Facility for Microscopy, Helmholtz Centre for Infection Research, Inhoffenstraße 7, 38124 Braunschweig, Germany; Robert Koch-Institute, GERMANY

## Abstract

*Salmonella enterica* utilizes flagellar motility to swim through liquid environments and on surfaces. The biosynthesis of the flagellum is regulated on various levels, including transcriptional and posttranscriptional mechanisms. Here, we investigated the motility phenotype of 24 selected single gene deletions that were previously described to display swimming and swarming motility effects. Mutations in *flgE*, *fliH*, *ydiV*, *rfaG*, *yjcC*, STM1267 and STM3363 showed an altered motility phenotype. Deletions of *flgE* and *fliH* displayed a non-motile phenotype in both swimming and swarming motility assays as expected. The deletions of STM1267, STM3363, *ydiV*, *rfaG* and *yjcC* were further analyzed in detail for flagellar and fimbrial gene expression and filament formation. A Δ*ydiV* mutant showed increased swimming motility, but a decrease in swarming motility, which coincided with derepression of curli fimbriae. A deletion of *yjcC*, encoding for an EAL domain-containing protein, increased swimming motility independent on flagellar gene expression. A ΔSTM1267 mutant displayed a hypermotile phenotype on swarm agar plates and was found to have increased numbers of flagella. In contrast, a knockout of STM3363 did also display an increase in swarming motility, but did not alter flagella numbers. Finally, a deletion of the LPS biosynthesis-related protein RfaG reduced swimming and swarming motility, associated with a decrease in transcription from flagellar class II and class III promoters and a lack of flagellar filaments.

## Introduction

The gram-negative enteropathogen *Salmonella enterica* is the causative agent of salmonellosis. Typical symptoms include gastroenteritis, including diarrhea, abdominal cramps and in rare cases enteric fever, which can be life threatening, especially for immune-compromised people [1]. After ingestion of contaminated food or water *Salmonella* employs a battery of virulence factors, such as toxin injection devices (injectisomes) [2,3], fimbriae or flagella to successfully colonize and persist within the host. The flagellum, a rotating, rigid, helical motility organelle, enables bacteria to chemotactically swim towards nutrients or away from harmful substances [4]. Furthermore, flagella facilitate biofilm formation and adherence to the host epithelium. By enabling bacteria-host interactions, flagella therefore enhance bacterial pathogenesis [5].

The flagellum consists of three main structures: the basal body, the hook and the filament. The basal body includes rotor and stator protein complexes necessary for motor-force generation and thereby flagellar rotation [6,7]. Additionally, a rod spans the periplasmic space from the inner membrane through the cell wall to the outer membrane. The hook extends from the cell surface to a length of approximately 55 nm and functions as a flexible linking structure between the membrane-embedded basal body and the rigid filament. The 10–15 μm long filament is built up of thousands of subunits of a single protein, flagellin (FljB or FliC in *Salmonella enterica*). Every flagellar subunit has to be exported to enable a proper assembly at the tip of the growing structure. For this purpose, *Salmonella* uses a flagellar-specific type-III secretion system (fT3SS) that is structurally and functionally related to the virulence-associated T3SS (vT3SS) of injectisome devices [8]. The secretion of flagellar components is proton-motive force-dependent and coupled to ATP hydrolysis and has to proceed in a timely and ordered fashion [9–11]. The flagellar regulon of *Salmonella enterica* serovar Typhimurium comprises more than 60 genes [12]. The biosynthesis of the flagellar apparatus relies on a hierarchical regulatory system, consisting of three flagellar promoter classes. On top of this hierarchy and under the control of a σ^70^-dependent class I promoter is the flagellar master operon *flhDC*. FlhD and FlhC form a heteromultimeric complex (FlhD_4_C_2_) that ultimately controls initiation of flagellar assembly [13,14]. Together with σ^70^/RNA polymerase, the FlhD_4_C_2_ complex activates transcription of genes that are under the control of class II promoters, resulting in the expression of proteins that make up the basal body and hook (HBB). The regulatory protein FlgM is expressed from a class II promoter and interacts with the flagellar-specific σ^28^-factor to prevent association of σ^28^ with RNA polymerase, which is needed for transcription from class III promoters. Upon completion of the hook-basal-body complex, the secretion mode of the fT3SS switches to export of late substrates. FlgM as a late secretion substrate is exported, which releases σ^28^ and results in expression of filament and chemosensory proteins, as well as motor force generators [15–18].

Regulation of flagellar biosynthesis occurs on various levels. A variety of environmental stimuli are integrated at the level of the class I promoter that ultimately determine expression of the master regulatory operon, *flhDC*. On a transcriptional level the cyclic AMP-catabolite activator protein (CAP) controls bacterial motility by activating the *flhDC* operon [19]. The histone-like nucleoid-structuring protein (H-NS) indirectly activates *flhDC* transcription by repressing a negative regulator, HdfR [20]. Transcription of *flhDC* is also activated by the iron-regulatory protein Fur [21] and the master regulator of the *Salmonella* pathogenicity island-1 encoded injectisome (Spi-1), HilD. HilD positively regulates flagellar gene expression through a direct binding to the P5 promoter of *flhDC* [22]. Furthermore, there are various negative regulators that bind to the *flhDC* promoter region, such as Spi-1 encoded RtsB, LrhA and SlyA [23–26]. On a posttranslational level, FlhD_4_C_2_ concentration is altered through FliZ, a flagellar protein that is under the control of class II and III promoters [27]. FliZ represses transcription of *ydiV* that encodes for a posttranscriptional anti-FlhD_4_C_2_ factor [28]. YdiV binds to FlhD, inhibits the DNA-binding properties of FlhD_4_C_2_ to class II promoters and targets the FlhD_4_C_2_ complex to ClpXP-mediated degradation [29,30].

Recently, Bogomolnaya et al. screened a library of 1023 single gene deletions in the virulent *Salmonella enterica* serovar Typhimurium background ATCC14028s for mutants that affected swimming and/or swarming motility [31,32]. Mutations in 130 genes that were not previously known to be involved in flagellar motility displayed an altered motility phenotype. Since various genes were linked to virulence in *Salmonella*, the flagellar system or were predicted to have a regulatory function, we were interested in the mechanisms of the altered motility phenotypes and investigated 24 presumed motility mutants in detail. We found that seven mutations (*flgE*, *fliH*, *ydiV*, *rfaG*, *yjcC*, STM1267, STM3363) affected swimming or swarming motility in our assays. A detailed analysis of flagella biosynthesis and flagellar gene expression for the mutant strains is described herein.

## Results

### Verification of novel factors modulating motility

**Fig 1:**
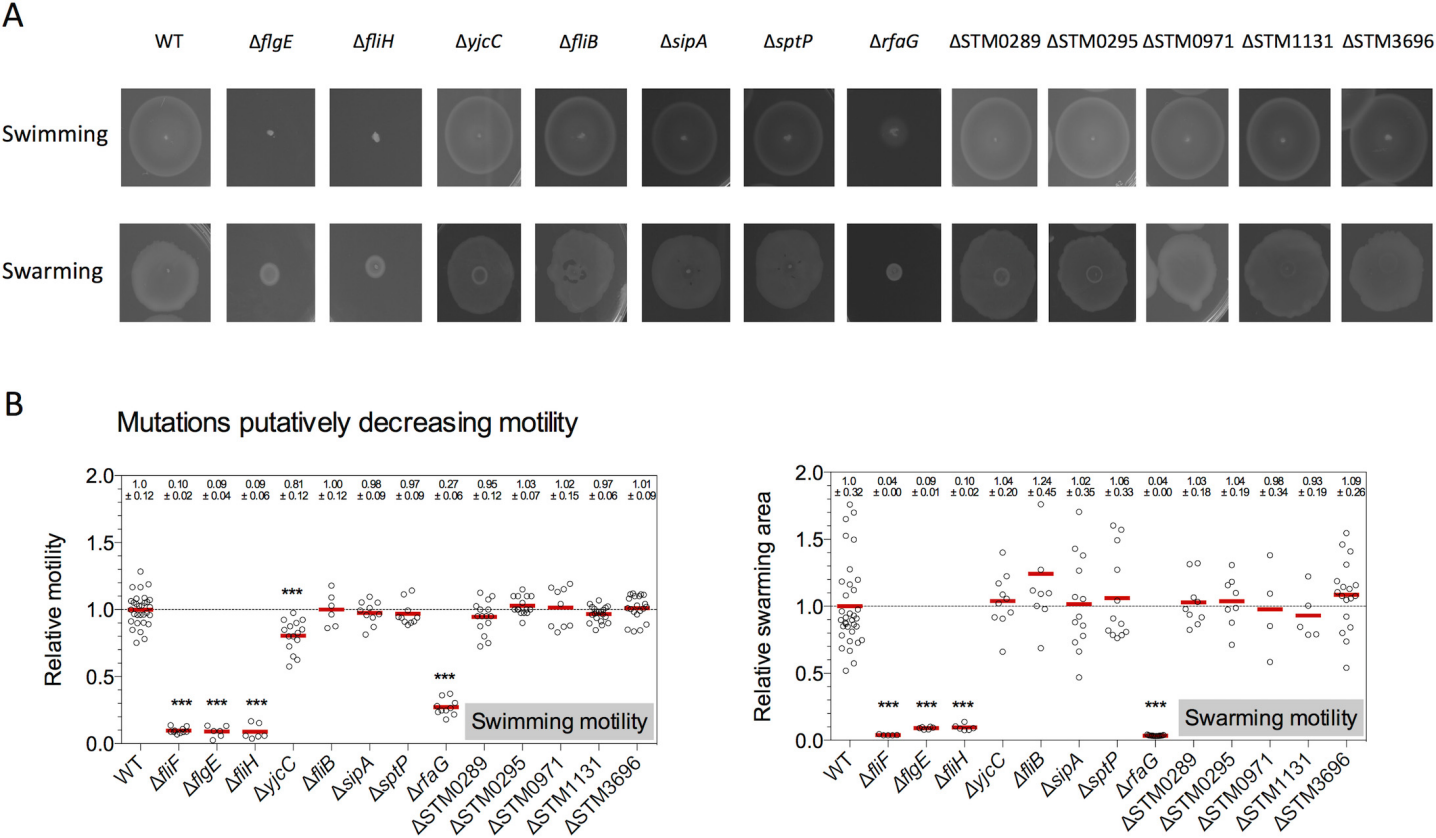
Swimming and swarming motility phenotypes of single gene deletion mutants putatively decreasing motility. Motility phenotypes of mutants were analyzed on soft-agar plates (0.3% agar) after 4 hours incubation at 37°C for swimming motility. Swarming motility was analyzed on plates containing 0.6% agar after 9 hours incubation at 37°C. (A) Representative motility plates for each mutant tested (TH6622, EM824, EM1480, EM1508, EM1511, EM1512, EM1688, EM1690, EM2384, EM2385, EM2590, EM2591, and EM2605). The diameter of the motility swarm and the swarming area were measured and normalized to the wildtype. (B) Quantified relative motility. Biological replicates are shown as individual data points and analyzed by the Student’s *t* test. Asterisks indicate a significantly different motility phenotype (*** = P<0.001).

**Fig 2:**
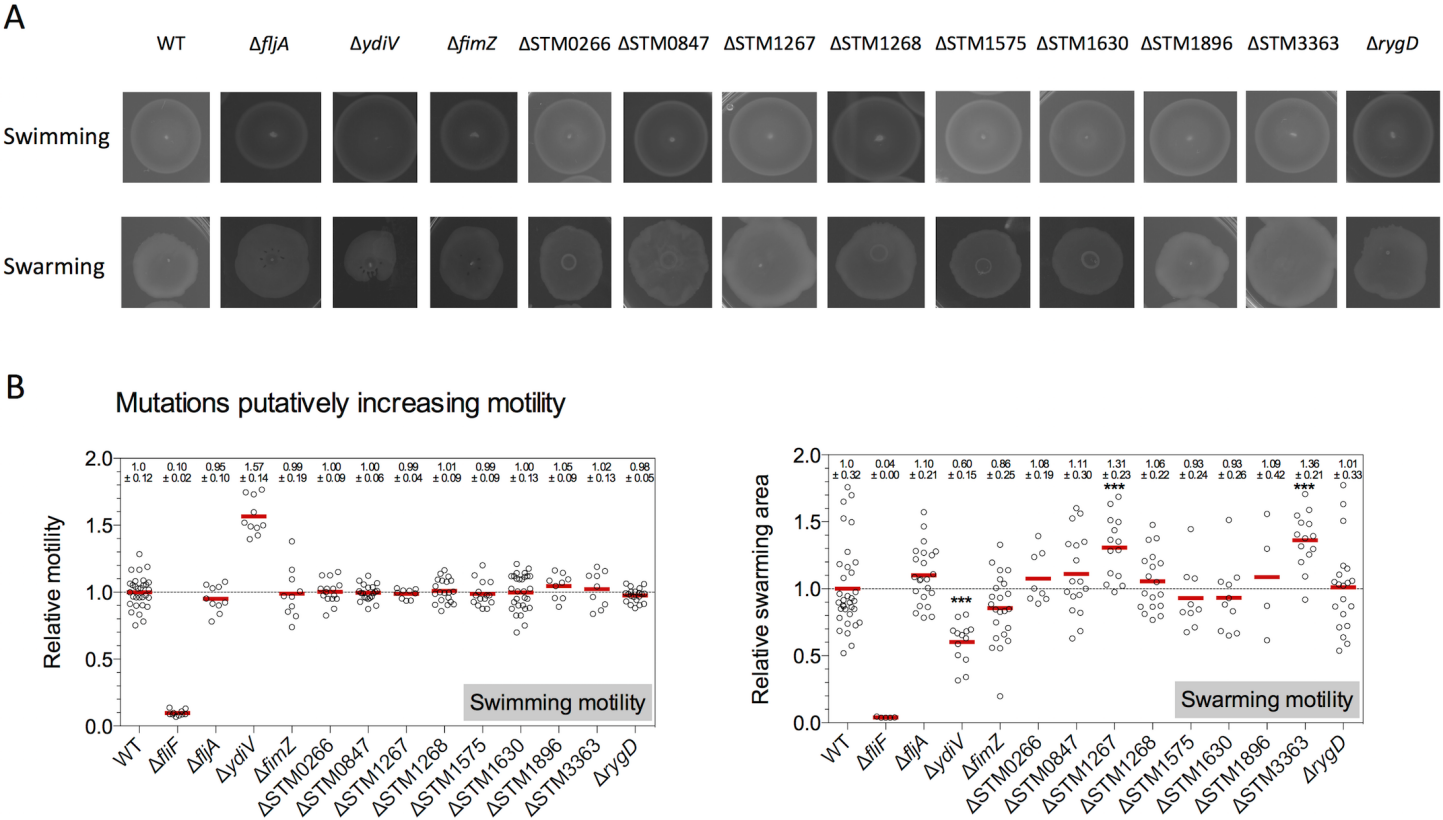
Swimming and swarming motility phenotypes of single gene deletion mutants putatively increasing motility. (A) Representative motility plates for mutants potentially increasing motility (TH6622, EM824, EM2381, EM2382, EM2383, EM1507, EM1686, EM1481, EM1689, EM1509, EM1510, EM1482, EM1484, and EM1691). The diameter of the motility swarm and the swarming area were measured and normalized to the wildtype. (B) Quantified relative motility. Biological replicates are shown as individual data points and analyzed by the Student’s *t* test. Asterisks indicate a significantly different motility phenotype (*** = P<0.001).

We constructed single gene deletions of putative factors modulating motility [31] using the λ-Red recombination system as described by Datsenko and Wanner [33]. We were particularly interested in genes that were linked to virulence of *Salmonella*, the flagellar system or were predicted to have a regulatory function. To verify the previously published motility phenotypes of selected putative motility regulators, we performed swimming and swarming motility assays. Of the 24 tested mutants, only seven (*flgE*, *fliH*, *ydiV*, *rfaG*, *yjcC*, STM1267, STM3363) displayed altered motility compared to the wildtype (WT) control (Figs 1 and 2 and summarized in S4 Table). This was independent of growth rates and doubling times (S1 Fig, S3 Table), the incubation time of the motility plates (S2 Fig) and the used *Salmonella enterica* strain (S3 Fig). A Δ*fliF* strain served as negative control.

In contrast to the swimming-only defect described in Bogomolnaya et al. [31], we found that *flgE* and *fliH* mutations resulted in a loss of both swimming and swarming motility (Fig 1). This was expected since FlgE, the flagellar hook protein, and FliH, a component of the fT3SS ATPase complex, are essential structural and functional components of the flagellum [34]. A deletion of *ydiV* showed increased swimming motility due to the posttranslational inhibition of flagellar class II gene expression via YdiV-mediated degradation of FlhD_4_C_2_ complex [29,30] (Fig 2). However, we found that swarming motility was decreased up to 50%. A deletion of *rfaG* showed a reduction in swimming (30% of WT) and a complete loss of swarming motility (Fig 1). Decreased swimming motility was also observed in a Δ*yjcC* mutant (80% of WT). Deletions of STM1267 and STM3363 –predicted genes with unknown functions—had no effect on swimming motility, but led to an increased swarming motility phenotype (Fig 2).

### Characterization of mutants with an altered motility phenotype

**Fig 3:**
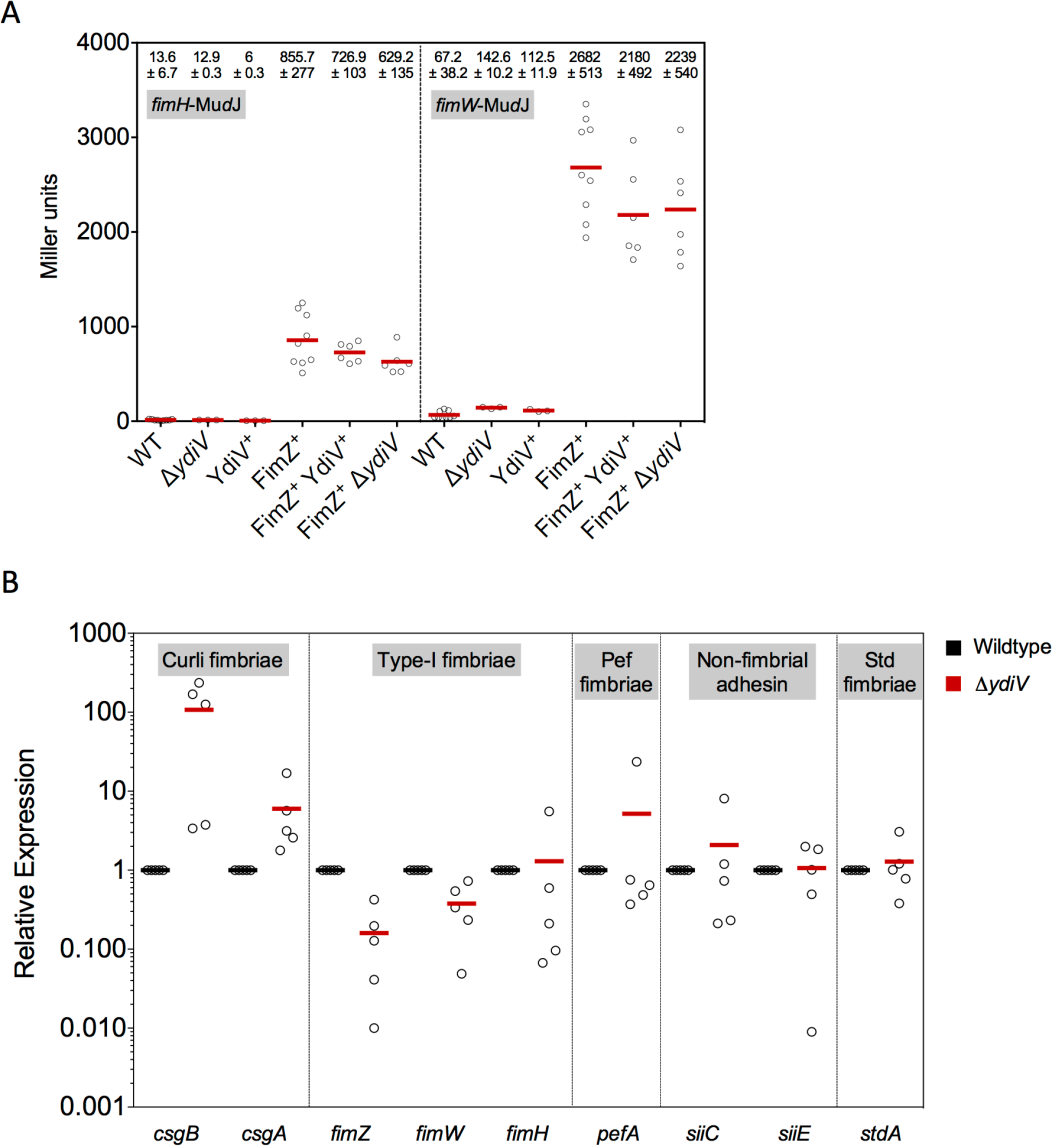
YdiV influences curli fimbriae gene transcription. (A) Effects of a Δ*ydiV* mutant and overproduced YdiV (YdiV^+^) and FimZ (FimZ^+^) on fimbrial gene expression were analyzed by β-galactosidase assay. Transcription of *fimH* that encodes a fimbrial structure component, and *fimW* that encodes for a repressor of fimbria, was examined in strains TH13438 (Δ*araBAD*997::*fimZ*+ *fimH*56::Mu*d*J), TH13439 (Δ*araBAD*997::*fimZ*+ *fimW*57::Mu*d*J), TH13498 (*fimH*56::Mu*d*J), TH13502 (Δ*araBAD*995::*ydiV*+ *fimH*56::Mu*d*J), TH13505 (*fimW*57::Mu*d*J), TH13509 (Δ*araBAD*995::*ydiV*+ *fimW*57::Mu*d*J), EM2527 (Δ*ydiV*252 *fimH*56::Mu*d*J), EM2528 (Δ*ydiV*252 *fimW*57::Mu*d*J), EM2724 (Δ*araBAD*997::*fimZ*+ *fimH*56::Mu*d*J *ydiV*240::Tn*10d*Tc[del-25]), EM2725 (Δ*araBAD*997::*fimZ*+ *fimW*57::Mu*d*J *ydiV*240::Tn*10d*Tc[del-25]), EM2726 (Δ*araBAD*997::*fimZ*+ *fimH*56::Mu*d*J Δ*ydiV*251::*tetRA*) and EM2727 (Δ*araBAD*997::*fimZ*+ *fimW*57::Mu*d*J Δ*ydiV*251::*tetRA*). At least three independent biological samples were analyzed. Error bars represent the standard errors of the means. Asterisks indicate gene expression levels that are significantly different to wildtype levels (*** = P< 0.001). (B) Relative *csgA*, *csgB*, *fimZ*, *fimW*, *fimH*, *pefA*, *siiC*, *siiE* and *stdA* gene expression of a Δ*ydiV*::FKF mutant (EM2382) compared to the wildtype (TH6622) as analyzed using qRT-PCR. Experiments were performed with 5 biological replicates.

We next characterized selected mutants that displayed effects on motility by analyzing transcription of fimbrial or flagellar genes and filament formation. Since the *ydiV* mutation decreased swarming ability, we hypothesized that YdiV might be involved in expression of fimbriae as shown for *Escherichia coli* [35] and thereby resulting in a stickier phenotype of the Δ*ydiV* deletion mutant in the swarm agar assay. Fimbrial gene transcription was analyzed by β-galactosidase activity assay using *lac* operon fusions to *fimH*, encoding for the fimbrial tip responsible for adhesion [36], and *fimW*, encoding the Type I fimbriae repressing protein FimW. A strain that overexpressed FimZ, a positive regulator of Type I fimbriae, was used as a control. Expression of FimZ led to higher transcription of *fimH* and *fimW* levels and these levels were neither influenced by a *ydiV* deletion, nor YdiV overexpression (Fig 3A), indicating that expression of Type I fimbriae was not affected by YdiV. *Salmonella enterica* serovar Typhimurium encodes for 13 fimbrial adhesins [37], and we next analyzed transcription of *csgAB*, *fimZ*, *pefA*, *siiC*, *siiE*, *stdA* adhesion genes by quantitative real-time PCR (Fig 3B). We found that in a Δ*ydiV* mutant gene expression of *csgA* and *csgB* was 3- and 100-fold increased, respectively, whereas none of the other fimbrial genes displayed significantly altered transcript levels.

A deletion of *yjcC* (STM4264), a putative phosphodiesterase with EAL-motif, showed decreased swimming motility, but had no effect on swarming motility. To investigate this motility decrease in more detail, we analyzed β-galactosidase activity in strains that harbored *lac*-fusions to flagellar class I (*flhC*), class II (*fliL*) and class III (*fliC*) promoters. Transcription levels of all flagellar gene fusions tested were similar to WT levels, suggesting that YjcC does not play a role in flagellar gene expression (S4 Fig).

**Fig 4:**
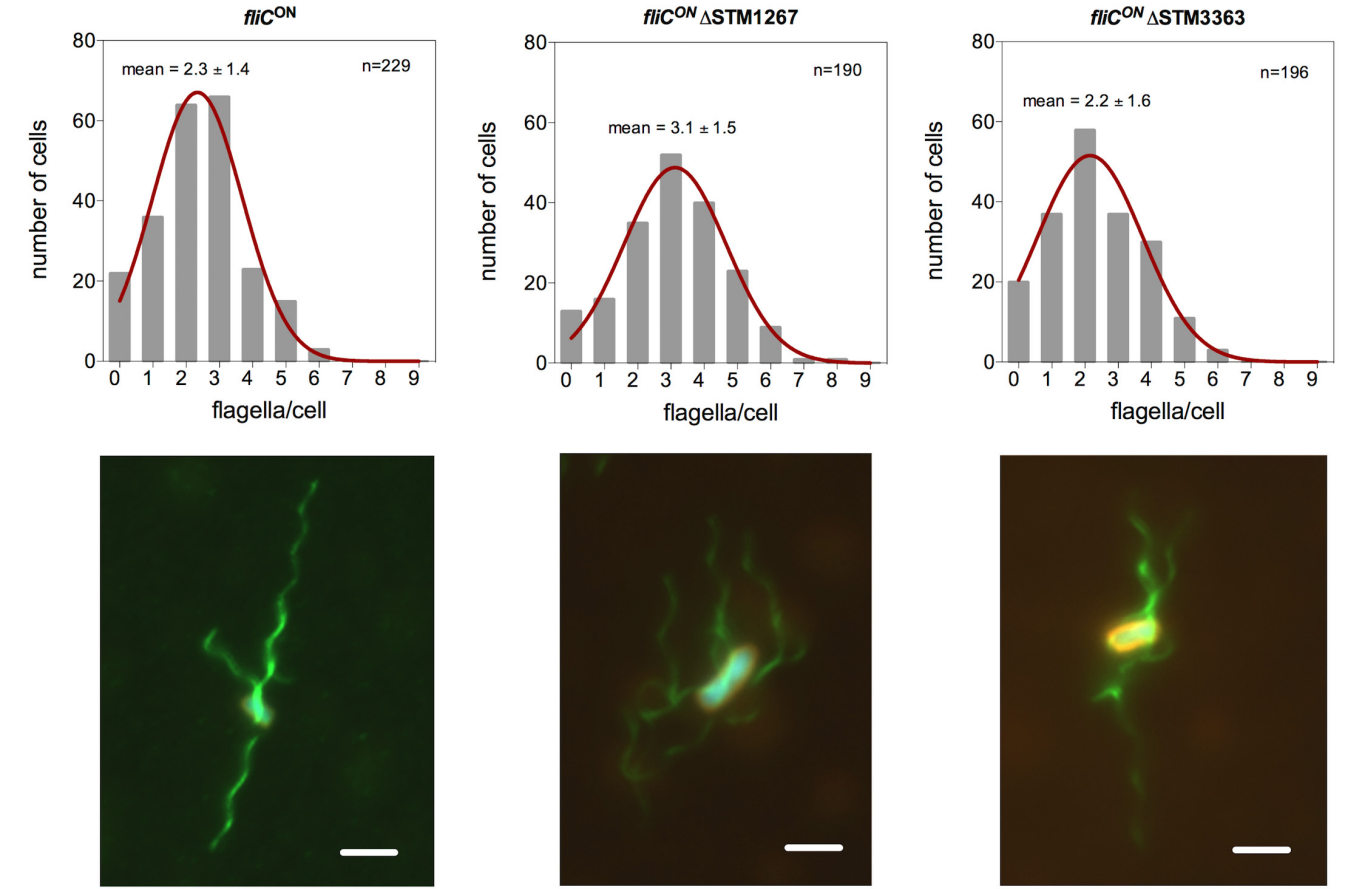
Flagellar filament formation is increased in a STM1267 deletion mutant. Top: Histogram of counted flagella per cell body of the wildtype, a STM1267::FRT and STM3363::FRT deletion mutant. The average filament numbers per cell based on a Gaussian non-linear regression analysis is indicated in the figure. Bottom: Flagellar filament formation was analyzed by flagellin immunostaining using α-FliC primary and α-rabbit conjugated Alexa Fluor 488 secondary antibodies (green) in a strain background locked for *fliC* expression. The membrane was stained with FM-64 (red) and DNA with DAPI (blue). Scale bar = 2 μm.

We next analyzed filament formation in two hypermotile mutants, ΔSTM1267 and ΔSTM3363, which showed an increased swarming motility phenotype. For that purpose, we analyzed flagellation levels by anti-FliC immunostaining in a strain locked in the FliC-phase. The WT strain assembled 2–3 flagella per cell (mean = 2.3 ± 1.4) under the experimental conditions (Fig 4). A ΔSTM3363 mutation did not affect filament formation (mean = 2.2 ± 1.6). A deletion in STM1267 slightly increased the number of flagella per cell (mean = 3.1 ± 1.5), which provided a possible explanation for the hypermotile phenotype.

**Fig 5:**
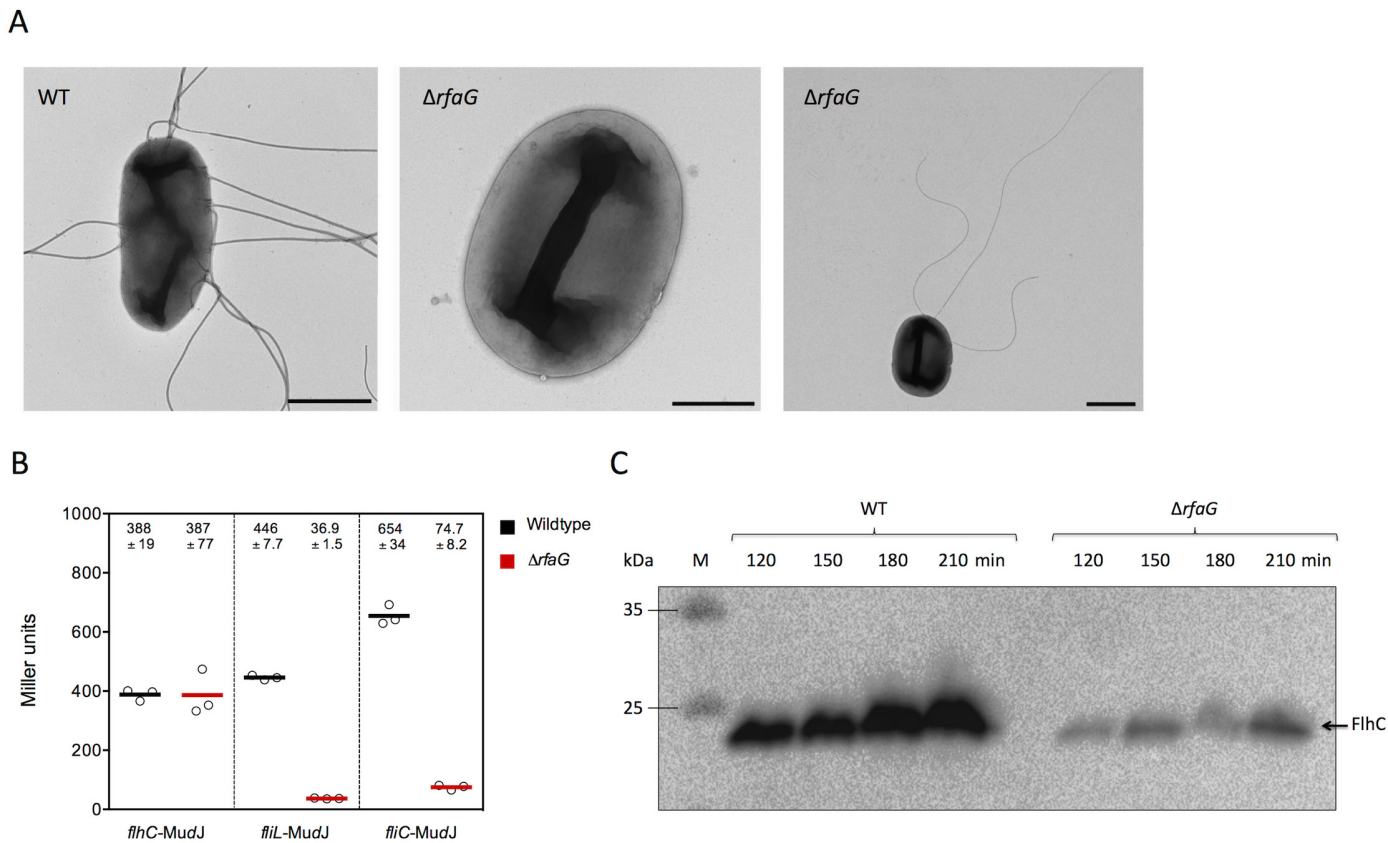
Flagella numbers and flagellar class II and class III gene expression are decreased in a Δ*rfaG* mutant. (A) Transmission electron microscopy (TEM) of wildtype and Δ*rfaG*::FRT strains. With the exception of few individual cells the Δ*rfaG*::FRT mutant is non-flagellated. Scale bars: Left and right panel = 1 μm; Middle panel = 0.5 μm. (B) Effect of Δ*rfaG*::FRT mutation on flagellar gene transcription from class I (*flhC*-Mu*d*J), class II (*fliL*-Mu*d*J) and class III (*fliC*-Mu*d*J) promoters. The β-galactosidase assay was performed in triplicate. Error bars represent the standard errors of the means. (C) FlhC-3xFLAG protein levels in the wildtype (EM1438) and Δ*rfaG* strain (EM2748). Bacteria were grown in LB medium and samples were taken 120, 150, 180 and 210 min after inoculation. Protein levels were monitored by SDS-PAGE and immunoblotting using monoclonal anti-FLAG antibodies.

Upon deletion of *rfaG*, which encodes for a glucosyltransferase, swimming motility was reduced up to 80% and swarming motility was abolished completely. Thus, we investigated flagellation of a Δ*rfaG* mutant by transmission electron microscopy. In comparison to the highly flagellated WT, we observed a primarily deflagellated phenotype (Fig 5A left and middle panels). Only few bacteria possessed a low number of flagella (Fig 5A right panel), which was in agreement with the remaining 20% of swimming motility. We hypothesized that RfaG regulated flagellar gene expression on a transcriptional level, which would explain the non-flagellated phenotype. We performed β-galactosidase assays with *lac*-fusions to flagellar class I (*flhC*), class II (*fliL*) and class III (*fliC*) promoters in a Δ*rfaG* deletion mutant. Transcription of *flhC* was not altered, however, a Δ*rfaG* mutation reduced transcription of class II and class III promoters significantly (Fig 5B). FlhC protein levels were examined using a 3x FLAG tagged version of FlhC [29]. FlhC protein levels were reduced in a *rfaG* deletion mutant in contrast to the WT (Fig 5C), indicating that RfaG is involved in regulation of flagellar class II gene expression by posttranscriptionally affecting FlhDC stability or mRNA translation.

## Discussion

Flagellar motility in *Salmonella enterica* serovar Typhimurium is of great importance in respect to biofilm formation, adherence and virulence. Thus, motility is tightly regulated and assembly of flagella occurs in an ordered fashion. There are various levels on which regulation of motility takes place: transcription of the master operon *flhDC* is regulated by a myriad of factors. Furthermore, FlhDC complex is subject to several posttranscriptional regulation mechanisms. In a recent genome-wide screen for novel motility regulators by Bogomolnaya et al., 130 mutations in the *Salmonella* Typhimurium genome were implicated to influence motility [31]. Here, we sought to characterize the molecular mechanisms of regulation of selected putative novel motility regulators. Seven of the 24 tested mutants showed an altered motility phenotype in our motility assays, which differ slightly in media composition and inoculation methods from the methodology used in Bogomolnaya et al. as described in detail in Materials and Methods.

For *flgE* and *fliH* mutations, it was described that only swimming motility was affected [31]. However, we found that Δ*flgE* and Δ*fliH* mutants were non-motile in both swimming and swarming motility agar assays. This result was expected, since FlgE (encoding for the hook protein) and FliH (encoding for an accessory protein of the flagellum-associated ATPase complex) are essential components of the flagellar structure and type-III export apparatus, respectively.

YdiV was shown previously to repress motility posttranslationally via targeted degradation of FlhD [29]. Accordingly, a *ydiV* deletion resulted in increased motility due to upregulation of FlhDC activity. However, we additionally observed a 50% decrease in swarming motility in the Δ*ydiV* mutant. For *Escherichia coli* it is known that YdiV represses Pap fimbriae [35]. Therefore, we hypothesized that in *Salmonella* YdiV might also affect fimbrial expression, leading to a stickier phenotype of the deletion mutant on swarming agar plates. However, type I fimbriae production was not upregulated in the Δ*ydiV* mutant indicating that YdiV is not involved in repression of type I fimbriae in *Salmonella*. 12 other fimbrial loci are predicted for *Salmonella enterica* serovar Typhimurium strain LT2, including curli fimbriae, Pef and Std fimbriae [37]. Here, we showed that a *ydiV* deletion led to increased mRNA levels of *csgA* and *csgB*, the major and minor subunits of curli fimbriae. However, in a recent study by Anwar et al. [38] a Δ*ydiV* deletion mutant showed reduced expression of CsgD, the master regulator of biofilm formation, and developed a reduced *rdar* (*r*ed, *d*ry *a*nd *r*ough) morphotype on congo red plates. This hints to a reduced expression of curli fimbriae. These contradictory results may be due to different media and growth conditions used. As described for *Myxococcus xanthus* or *Pseudomonas aeruginosa*, type IV pili play a role in twitching and gliding motility [39]. We thus suggest that the decreased swarming phenotype of the Δ*ydiV* mutant is due to derepression of curli fimbriae.

We next validated the influence of YjcC on swimming motility as described by Bogomolnaya et al. [31] and observed that YjcC does not affect flagellar gene expression in *Salmonella*. YjcC is a putative phosphodiesterase containing an EAL-motif (Glu-Ala-Leu). EAL-domain containing proteins degrade of cyclic di(3’→5’)-guanylic acid (c-di-GMP) [40]. C-di-GMP levels are known to influence many mechanisms, e.g. virulence, biofilm formation and motility. In *Salmonella* Typhimurium and *Escherichia coli*, motility is regulated through c-di-GMP on a posttranslational level via binding to receptors, including the cellulose synthase BcsA and the c-di-GMP binding protein YcgR [41,42]. Upon binding of c-di-GMP to YcgR, the loose interaction of YcgR with flagellar motor proteins is tightened, resulting in a slow down of flagellar rotation [41,43]. It has been described that a knockout of another EAL-containing protein YhjH (STM3611) reduced swarming motility [44] and that a Δ*yjcC* mutant exhibits higher c-di-GMP levels [40]. A deletion mutant of *yjcC* was shown to increase expression of curli fimbriae and cellulose biosynthesis, leading to higher adhesion to glass surfaces, which would provide a possible mechanism for the decrease of motility in the Δ*yjcC* mutant [40].

The genes STM1267 and STM3363 are not yet characterized in detail, but are conserved among *Salmonella* species. We showed that both knockouts of STM1267 and STM3363 increased swarming, but not swimming motility. STM3363 encodes for a hypothetical protein that contains a conserved Barstar-like domain, which was shown to play a role in a toxin-antitoxin system in *Bacillus subtilis* [45]. STM1267 encodes for a hypothetical protein with a conserved domain of the YmgB superfamily. In *Escherichia coli*, YmgB, also called AriR, represses biofilm formation and decreases cellular motility [46]. We demonstrated that a deletion of STM3363 increased swarming motility independently on filament formation, whereas a ΔSTM1267 mutant showed a higher number of flagella per cell body, which could account for the increased swarming motility phenotype. We cannot exclude that a deletion of STM3363 affected the recently described PagM-mediated flagella-independent surface motility [47].

Finally, we showed a severe defect of a Δ*rfaG* mutant in both swimming and swarming motility. RfaG is involved in biosynthesis of lipopolysaccharides (LPS), connecting inner and outer core. In particular, the O-antigen of LPS has been reported to contribute to swarming motility for several gram-negative bacteria [48–51]. The core and O-antigen polysaccharide of LPS confers hydrophilicity to the cell surface and in addition, LPS reduces the surface tension between the agar and cell surface (reviewed in [52]). In *Escherichia coli* it was shown previously that genes involved in biosynthesis of LPS are required for swarming, but not for swimming motility [53]. It was described by Raetz et al. that knockouts of proteins responsible for the construction of the inner core also impaired swimming motility [54], suggesting that flagellar assembly and function may be influenced by altered LPS structures. Here, we show that a Δ*rfaG* deletion diminished flagellar assembly and significantly reduced transcription of flagellar class II and class III promoters, but not of the class I promoter. Moreover, FlhC protein levels were reduced in the Δ*rfaG* strain. We conclude that a defect in LPS biosynthesis regulates motility by affecting FlhDC stability or translation of its mRNA on a posttranscriptional level via an unknown mechanism.

In summary, we analyzed in this study the molecular mechanisms by which recently described novel factors modulate motility [31]. We were able to verify the published motility effects of seven of 24 selected putative motility regulators. We hypothesize that a combination of several factors might explain the lack of motility phenotype for several other putative novel motility factors. First, it appears possible that off-site effects of the λ-RED-mediated construction of the single deletion mutants might result in false-positive hits. To exclude secondary site mutations, it is advisable to re-transduce gene deletions constructed by λ-RED-mediated recombination into a clean background. Bogomolnaya et al. [31] did not perform a re-transduction of the deletion mutants of the high-throughput screen, but the authors advised such a procedure for follow-up studies. Second, in Bogomolnaya et al. an antibiotic resistance cassette was inserted in the location of the deleted gene, whereas in this study, the antibiotic resistance cassettes were removed for deletions in *ydiV*, *fliB*, STM0971, STM1267, STM1896, STM3363, STM0266, STM0295, STM1575, STM1630, *yjcC*, STM0289, STM0847, STM1131, STM1268, STM3696, *rygD*, *flgE*, *fliH*, and *rfaG* in order to minimize the possibility of polar effects. It appears possible that some of the motility effects observed by Bogomolnaya et al. were indirect due to polar effects of the inserted antibiotic resistance cassette. Third, the motility assays in Bogomolnaya et al. were performed using different media compositions for swimming motility plates (e.g. the motility plates of Bogomolnaya et al. contained yeast extract), inoculation methods and time points for quantification when compared to the methodology used here (see also Materials and Methods). It is possible that certain motility phenotypes are specific for the conditions and media composition used in Bogomolnaya et al. and it is also a possibility that—due to the high-throughput screening format of Bogomolnaya et al.–the quantification of small swimming and swarming motility areas might have lead to misinterpretation. Swarming motility is highly influenced by humidity and wetness that may contribute to alterations in motility phenotypes.

However, importantly, we were able to identify possible regulatory mechanisms of several novel factors that were originally described by Bogomolnaya et al. [31] to be involved in motility regulation, in particular of STM1267, STM3363, RfaG, YdiV and YjcC. Among the novel factors implicated by Bogomolnaya et al. [31] to be involved in swimming and swarming motility are many additional genes whose functions are currently unknown or poorly understood. We conclude that careful examination of the described motility defects is needed by independently constructed deletion mutants, but further studies on these genes have great potential to lead to a better understanding of the molecular mechanisms involved in motility of *Salmonella*.

## Materials and Methods

### Bacterial strains, plasmids and media

All bacterial strains used in this study are listed in S1 Table and were derived from ATCC14028s. Cells were grown in lysogeny broth (LB) at 37°C, whereas strains harboring a temperature-sensitive λ-RED plasmid were grown at 30°C [55]. Bacterial growth was measured via optical density at 600 nm in a Varioskan Flash plate reader (Thermo Scientific). Motility agar was prepared and swimming motility was examined as described before [56]. Swarming motility of overnight cultures was assayed on 0.6% Difco Bacto Agar (LB Miller base 25 g/l, 0.5% glucose) in closed containers with 100% humidity. The generalized transducing phage of *Salmonella enterica* serovar Typhimurium P22 *HT105*/*1 int-201* was used in all transductional crosses [57]. Single gene deletions of genes *ydiV*, *fliB*, STM0266, STM0295, STM1575, STM1630, *yjcC*, STM1512, *fljA*, *fimZ*, *sipA*, *sptP*, *flgE*, and *fliH* were produced as previously described [33] and retained 10 codons of the respective coding region (15 bp each at either the 5’ and 3’ ends) in addition to the FRT scar after removal of the antibiotics resistance cassette. The complete coding region was deleted for genes STM3696, STM1131, STM0847, STM1268, STM0971, STM1267, STM1896 and STM3363. The *rfaG* mutation was inserted as described in [58]. The oligonucleotides used for strain construction are listed in S2 Table.

### β-galactosidase assays

β-galactosidase activity was measured as described before using at least three independent biological replicates [59]. Cultures were supplemented with 0.2% arabinose if required for the induction of P_*araB*_ expression or 0.1 μg/ml anhydrotetracyclin to induce *tetA*-dependent expression. For fimbrial gene expression studies the cultures were grown under static growth conditions.

### RNA isolation and quantitative real-time PCR

Strains were grown under static growth conditions in LB medium and total RNA isolation was performed using the RNeasy minikit (Qiagen). For removal of genomic DNA, RNA was treated with DNase using the TURBO DNA-free kit (ambion). Reverse transcription and quantitative real-time PCRs (qRT-PCR) were performed using the SensiFast SYBR No-ROX One Step kit (Bioline) in a Rotor-Gene Q lightcycler (Qiagen). Relative changes in mRNA levels were analyzed according to Pfaffl [60] and normalized against the transcription levels of multiple reference genes according to the method described by Vandesompele et al. [61]. The reference genes *gyrB*, *gmk* and *rpoD* were used as previously described [22, 59].

### Fluorescent microscopy

Fluorescent microscopy analysis was performed as described before [62]. Logarithmically growing bacteria were fixed onto poly-L-lysine pre-coated coverslips by addition of 2% formaldehyde and 0.2% glutaraldehyde. Flagella were stained using polyclonal anti-FliC antibodies (rabbit) and anti-rabbit conjugated Alexa Fluor 488 secondary antibodies (Invitrogen). The cell membranes were stained using FM-64 (0.5 mg/ml, Invitrogen) and DNA staining was performed using DAPI (Sigma-Aldrich). Images were taken using an Axio Observer microscope with an Axiocam HR camera (Zeiss) at 1000x magnification. Images were analyzed and contrast and brightness were adjusted using ImageJ (http://rsbweb.nih.gov/ij/).

### Electron microscopy

Strains were cultured in 5 ml LB or minimal media overnight. 650 μl glutaraldehyde (2%) was added to fixate the bacteria. The mixture was stored in the fridge at 4°C. For TEM observation bacteria were negatively stained with 2% aqueous uranyl acetate using a carbon film deposited on mica. Samples were examined in a Zeiss TEM 910 at an acceleration voltage of 80 kV with calibrated magnifications. Images were recorded digitally with a Slow-Scan CCD-Camera (ProScan, 1024 x 1024) with ITEM-Software (Olympus Soft Imaging Solutions). Contrast and brightness were adjusted with Adobe Photoshop CS3.

### Analysis of protein levels

A single colony of the analyzed strains was cultured in LB medium overnight, diluted 1:100 into fresh LB medium and grown for 120–210 min. 2 ml samples of the culture were taken every 30 min and whole cell lysates were prepared by normalizing the samples to measured OD600 values using SDS sample buffer. Protein levels were analyzed by SDS polyacrylamide gel electrophoresis and immunoblotting using anti-FLAG antibodies (Sigma).

## Supporting Information

S1 Fig Growth curves of single gene deletion mutants. Growth was measured via absorption at 600 nm every 15 minutes for 13 h with at least three biological replicates of each mutant strain (EM880, EM1480, EM1481, EM1482, EM1484, EM1507, EM1508, EM1509, EM1510, EM1511, EM1512, EM1686, EM1688, EM1689, EM1690, EM1691, EM2381, EM2382, EM2383, EM2384, EM2385, EM2590, EM2591 and EM2605). The dotted lines represent the standard error of the mean.
(TIF)

S2 Fig Analysis of swimming motility dependent on incubation time. Swimming motility of single gene deletion mutants in STM1896 (EM1482 ΔSTM1896::FRT), STM1630 (EM1510 ΔSTM1630::FRT) and STM3696 (EM1690 ΔSTM3696::FRT) was monitored over time to exclude incubation time dependent swimming behaviors. The motility assay was performed with four biological replicates.
(TIF)

S3 Fig Swimming motility of various *Salmonella* strains. The *Salmonella enterica* serovar Typhimurium strains ATCC14028s, SL1344, LT2 and SJW1103 (TH6622, EM774, TH437 and TH8362) were examined on swimming motility agar. The diameter of the motility swarm was measured and normalized to ATCC14028s. Biological replicates are shown as individual data points.
(TIF)

S4 Fig YjcC does not modulate swimming motility on a transcriptional level. Transcription of class I (*flhC*-Mu*d*J), class II (*fliL*-Mu*d*J) and class III (*fliC*-Mu*d*J) promoters were monitored for wildtype strains (EM584, EM2586 and EM2585) and Δ*yjcC*::FRT mutants (EM2587, EM2588 and EM2589) by β-galactosidase assay. Three independent biological samples were analyzed. Error bars represent the standard errors of the means.
(TIF)

S1 Table *Salmonella enterica* serovar Typhimurium ATCC14028s strains used in this study. (DOCX)

S2 Table Oligonucleotide sequences used for construction of single gene deletions in *Salmonella* and qRT-PCR assays. (DOCX)

S3 Table Doubling times and growth rates of single gene deletion mutants. (DOCX)

S4 Table Motility phenotypes of single gene deletion mutants. Significantly increased or decreased motility phenotypes are shown in bold.
(DOCX)
