# Supplementary figures and images for: Characterization of Novel Factors Involved in Swimming and Swarming Motility in Salmonella enterica Serovar Typhimurium
Source: PLoS One. 2015 Aug 12;10(8):e0135351. doi: 10.1371/journal.pone.0135351 (PMC4534456; doi:10.1371/journal.pone.0135351)

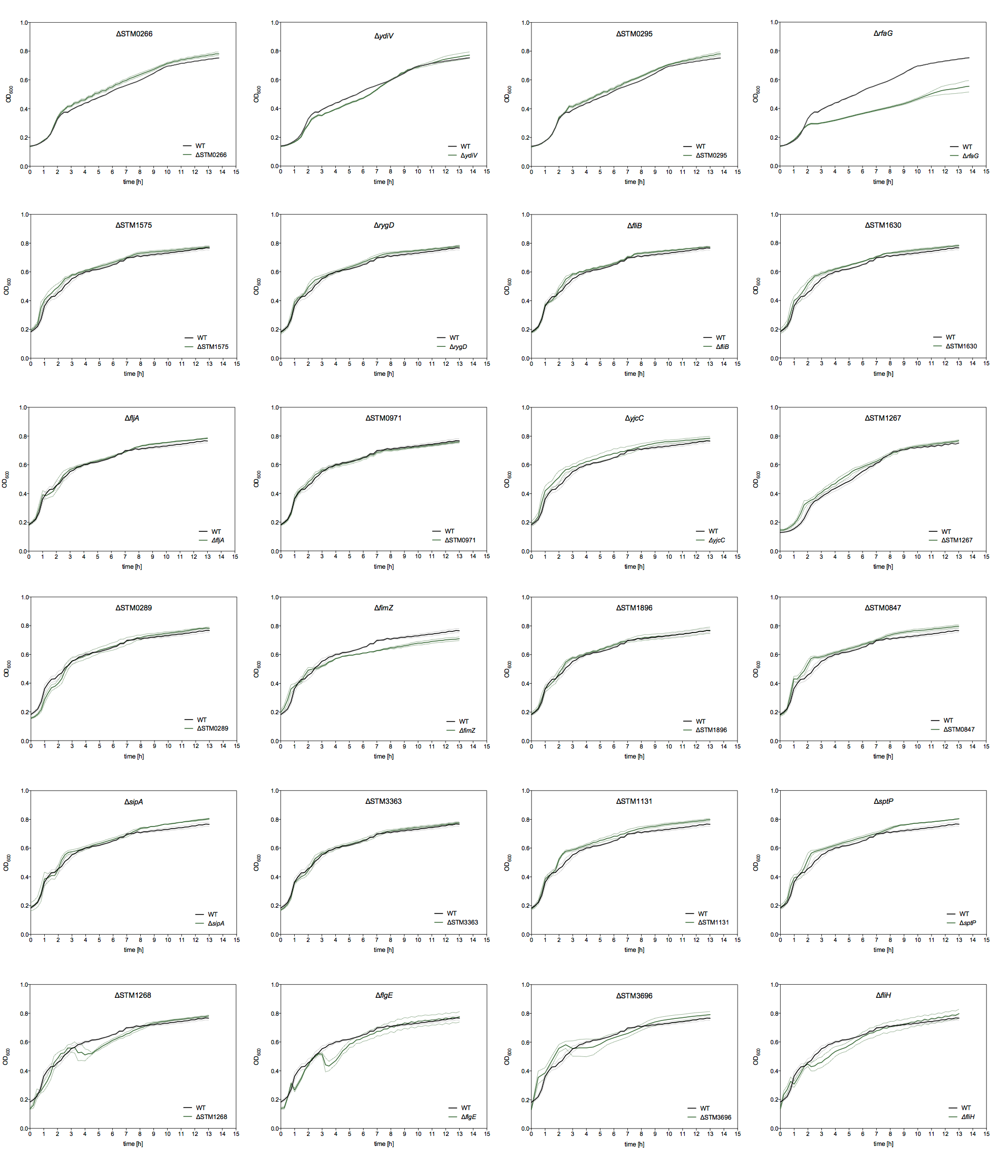

Supplement: S1 Fig — Growth was measured via absorption at 600 nm every 15 minutes for 13 h with at least three biological replicates of each mutant strain (EM880, EM1480, EM1481, EM1482, EM1484, EM1507, EM1508, EM1509, EM1510, EM1511, EM1512, EM1686, EM1688, EM1689, EM1690, EM1691, EM2381, EM2382, EM2383, EM2384, EM2385, EM2590, EM2591 and EM2605). The dotted lines represent the standard error of the mean. (TIF) [file pone.0135351.s001.tif]

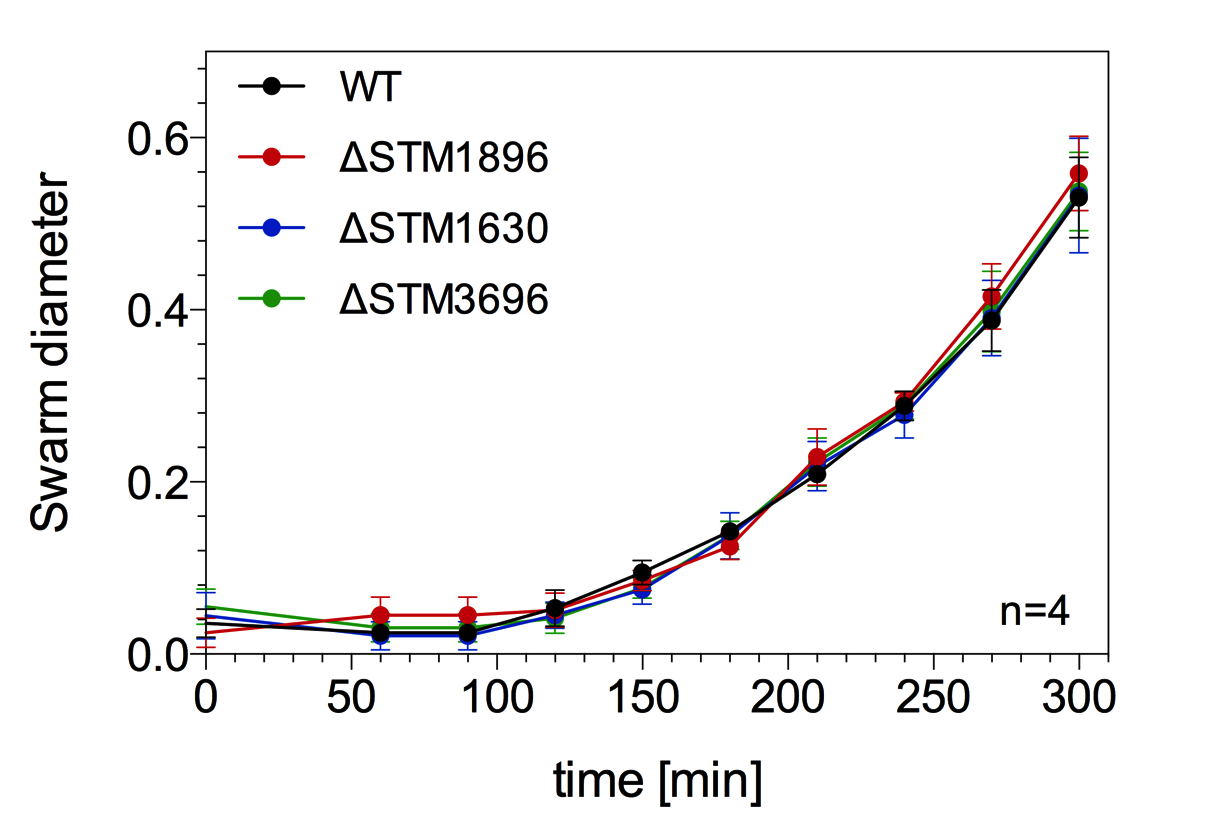

Supplement: S2 Fig — Swimming motility of single gene deletion mutants in STM1896 (EM1482 ΔSTM1896::FRT), STM1630 (EM1510 ΔSTM1630::FRT) and STM3696 (EM1690 ΔSTM3696::FRT) was monitored over time to exclude incubation time dependent swimming behaviors. The motility assay was performed with four biological replicates. (TIF) [file pone.0135351.s002.tif]

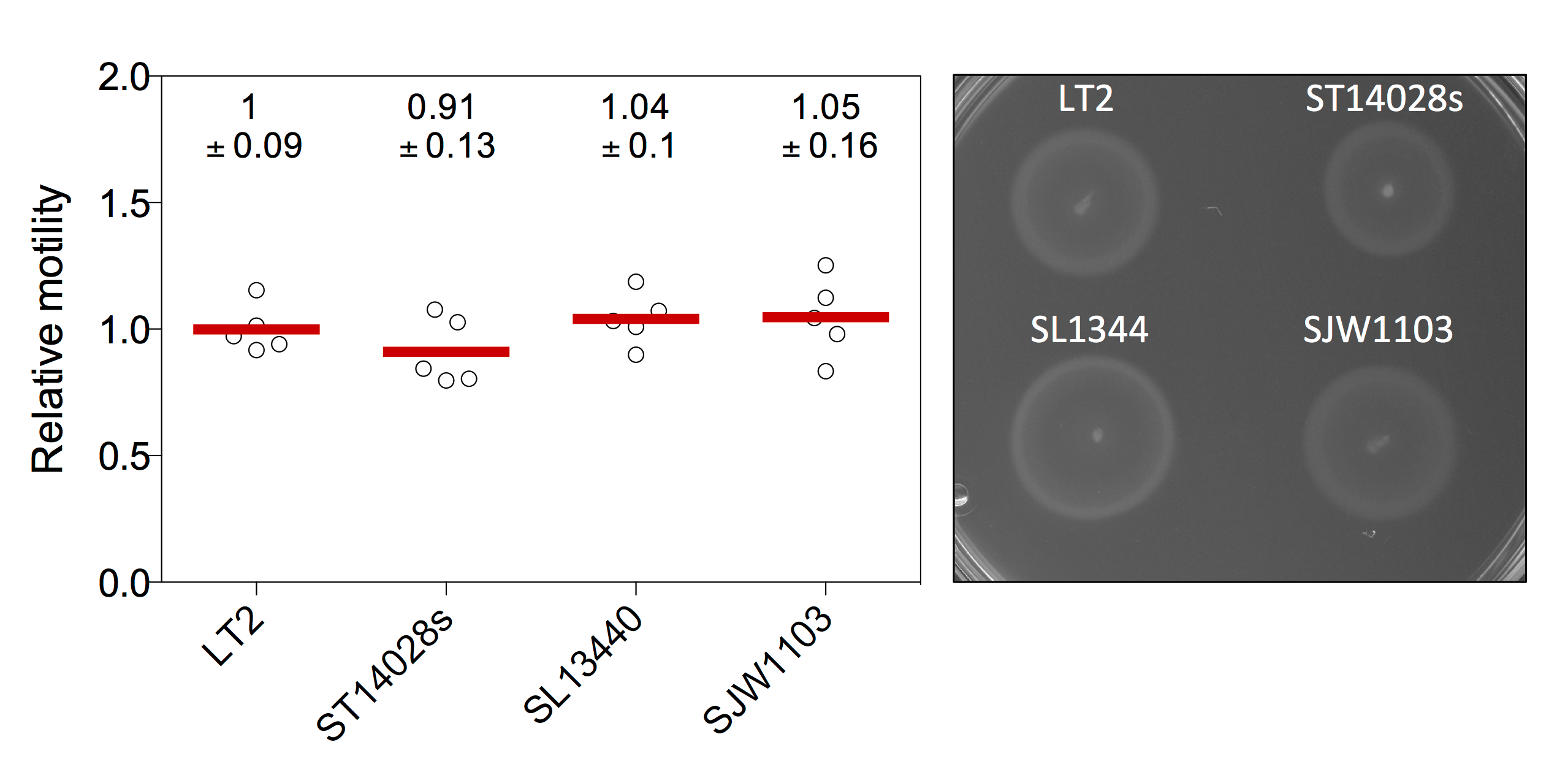

Supplement: S3 Fig — The Salmonella enterica serovar Typhimurium strains ATCC14028s, SL1344, LT2 and SJW1103 (TH6622, EM774, TH437 and TH8362) were examined on swimming motility agar. The diameter of the motility swarm was measured and normalized to ATCC14028s. Biological replicates are shown as individual data points. (TIF) [file pone.0135351.s003.tif]

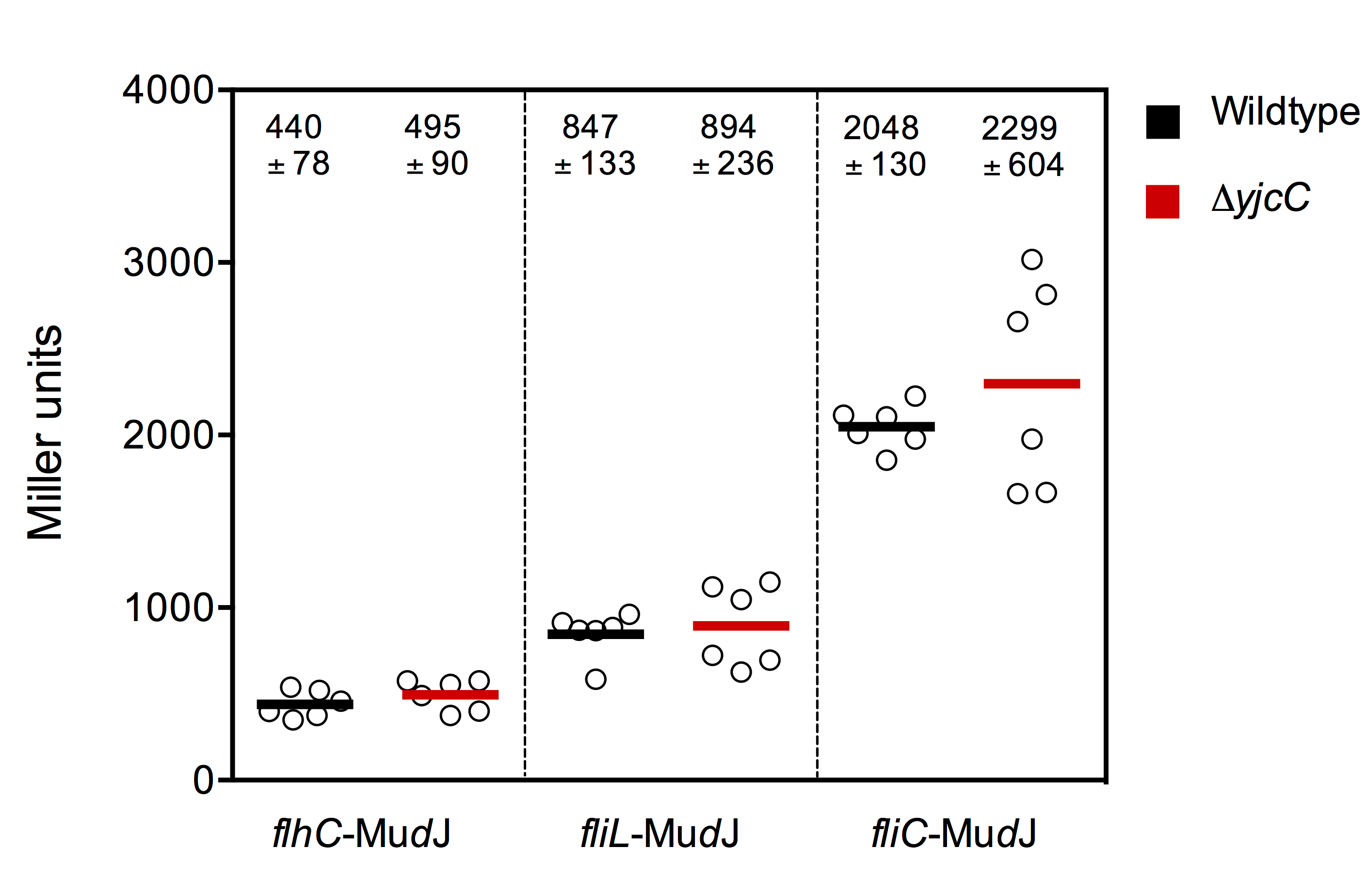

Supplement: S4 Fig — Transcription of class I (flhC-MudJ), class II (fliL-MudJ) and class III (fliC-MudJ) promoters were monitored for wildtype strains (EM584, EM2586 and EM2585) and ΔyjcC::FRT mutants (EM2587, EM2588 and EM2589) by β-galactosidase assay. Three independent biological samples were analyzed. Error bars represent the standard errors of the means. (TIF) [file pone.0135351.s004.tif]
