# Supplementary material for: Characterization of Novel Factors Involved in Swimming and Swarming Motility in Salmonella enterica Serovar Typhimurium
Source: PLoS One. 2015 Aug 12;10(8):e0135351. doi: 10.1371/journal.pone.0135351 (PMC4534456; doi:10.1371/journal.pone.0135351)
Supplement: S1 Table — (DOCX) [file pone.0135351.s005.docx]

**Supplementary Material:**

**Table S1:**

*Salmonella enterica* serovar Typhimurium ATCC14028s strains used in this study.

| **Strains** | **Relevant characteristics** | **Reference or source** |
| --- | --- | --- |
| TH437 | LT2 WT | Kelly T. Hughes |
| TH6622 | ATCC14028s WT | Kelly T. Hughes |
| TH8362 | SJW1103 WT | Kelly T. Hughes |
| TH11254 | Δ*hin*-5717::FRT (*fliC*^ON^) | Kelly T. Hughes |
| TH13438 | Δ*araBAD*997::*fimZ*+ *fimH*56::Mu*d*J | Kelly T. Hughes |
| TH13439 | Δ*araBAD*997::*fimZ*+ *fimW*57::Mu*d*J | Kelly T. Hughes |
| TH13498 | *fimH*56::Mu*d*J | Kelly T. Hughes |
| TH13502 | Δ*araBAD*995::*ydiV*+ *fimH*56::Mu*d*J | Kelly T. Hughes |
| TH13505 | *fimW*57::Mu*d*J | Kelly T. Hughes |
| TH13509 | Δ*araBAD*995::*ydiV*+ *fimW*57::Mu*d*J | Kelly T. Hughes |
| EM584 | *flhC*5213::Mu*d*J | This study |
| EM774 | SL1344 WT | Michael Kolbe |
| EM824 | Δ*fliF*7355 | Lab collection |
| EM880 | Δ*fliB*8191 | This study |
| EM1438 | *flhC*::3xFLAG-FRT | This study |
| EM1480 | ΔSTM0971::FRT | This study |
| EM1481 | ΔSTM1267::FRT | This study |
| EM1482 | ΔSTM1896::FRT | This study |
| EM1484 | ΔSTM3363:FRT | This study |
| EM1507 | ΔSTM0266::FRT | This study |
| EM1508 | ΔSTM0295::FRT | This study |
| EM1509 | ΔSTM1575::FRT | This study |
| EM1510 | ΔSTM1630::FRT | This study |
| EM1511 | Δ*yjcC*::FRT | This study |
| EM1512 | ΔSTM0289::FRT | This study |
| EM1686 | ΔSTM0847::FRT | This study |
| EM1688 | ΔSTM1131::FRT | This study |
| EM1689 | ΔSTM1268::FRT | This study |
| EM1690 | ΔSTM3696::FRT | This study |
| EM1691 | Δ*rygD*::FRT (deletes sRNA) | This study |
| EM1780 | ΔSTM1267::FRT *flhC*5213::Mu*d*J | This study |
| EM1781 | ΔSTM1267::FRT *fliL*5100::Mu*d*J | This study |
| EM1783 | ΔSTM3363::FRT *flhC*5213::Mu*d*J | This study |
| EM1784 | ΔSTM3363::FRT *fliL*5100::Mu*d*J | This study |
| EM2375 | ΔSTM1267::FRT Δ*hin*-5717::FCF | This study |
| EM2376 | ΔSTM3363::FRT Δ*hin*-5717::FCF | This study |
| EM2381 | Δ*fljA*5576::FKF | This study |
| EM2382 | Δ*ydiV*::FKF | This study |
| EM2383 | Δ*fimZ*::FKF | This study |
| EM2384 | Δ*sipA*::FKF | This study |
| EM2385 | Δ*sptP*::FKF | This study |
| EM2476 | ΔSTM1267::FRT Δ*hin*-5717::FCF *fliC*5050::Mu*d*J | This study |
| EM2477 | ΔSTM3363::FRT Δ*hin*-5717::FCF *fliC*5050::Mu*d*J | This study |
| EM2527 | Δ*ydiV*252 *fimH*56::Mu*d*J | This study |
| EM2528 | Δ*ydiV*252 *fimW*57::Mu*d*J | This study |
| EM2585 | Δ*hin*-5717::FCF *fliC*5050::Mu*d*J | This study |
| EM2586 | *fliL*5100::Mu*d*J | This study |
| EM2587 | Δ*yjcC*::FRT Δ*hin*-5717::FCF *fliC*5050::Mu*d*J | This study |
| EM2588 | Δ*yjcC*::FRT *fliL*5100::Mu*d*J | This study |
| EM2589 | Δ*yjcC*::FRT *flhC*5213::Mu*d*J | This study |
| EM2590 | Δ*flgE*7659 | This study |
| EM2591 | Δ*fliH*7363 | This study |
| EM2605 | Δ*rfaG*::FRT | (1) |
| EM2606 | Δ*rfaG* *flhC*5213::Mu*d*J | This study |
| EM2607 | Δ*rfaG* *fliL*5100::Mu*d*J | This study |
| EM2608 | Δ*rfaG* Δ*hin*-5718::FCF *fljB*5001::Mu*d*J | This study |
| EM2609 | Δ*hin*-5718::FRT *fljB*5001::Mu*d*J | This study |
| EM2724 | Δ*araBAD*997::*fimZ*+ *fimH*56::Mu*d*J *ydiV*240::Tn*10d*Tc[del-25] | This study |
| EM2725 | Δ*araBAD*997::*fimZ*+ *fimW*57::Mu*d*J *ydiV*240::Tn*10d*Tc[del-25] | This study |
| EM2726 | Δ*araBAD*997::*fimZ*+ *fimH*56::Mu*d*J Δ*ydiV*251::*tetRA* | This study |
| EM2727 | Δ*araBAD*997::*fimZ*+ *fimW*57::Mu*d*J Δ*ydiV*251::*tetRA* | This study |
| EM2748 | *flhC*::3xFLAG-FRT Δ*rfaG* | This study |

**Reference:**

1. Zenk SF, Jantsch J, Hensel M. Role of *Salmonella enterica* lipopolysaccharide in activation of dendritic cell functions and bacterial containment. J. Immunol. 2009; 183:2697–707.
