# Supplementary material for: Characterization of Novel Factors Involved in Swimming and Swarming Motility in Salmonella enterica Serovar Typhimurium
Source: PLoS One. 2015 Aug 12;10(8):e0135351. doi: 10.1371/journal.pone.0135351 (PMC4534456; doi:10.1371/journal.pone.0135351)
Supplement: S2 Table — (DOCX) [file pone.0135351.s006.docx]

**Table S2:**

Oligonucleotide sequences used for construction of single gene deletions in *Salmonella* and qRT-PCR assays.

| **Primer name** | **5’-3’ sequence** |
| --- | --- |
| 5'-STM3696-delFRT_fw | TGCGCGGATAAATCAGCCAATCTTTGCAGGGGCATCAGAC**GTGTAGGCTGGAGCTGCTTC** |
| 5'-STM3696-delFRT_rv | GCCTGATCCGGCCTGGCCGATCCCGATGGCATGTTCCCGT**CATATGAATATCCTCCTTAG** |
| 5'-STM1131-delFRT_fw | TAAATACAAAAATAAAGTTAATTGATGAGCGGAGTATTTT**GTGTAGGCTGGAGCTGCTTC** |
| 3'-STM1131-delFRT_rv | ATACTAAGCAAAGATTAGTACAGTAACTCTTTAACCTTTC**CATATGAATATCCTCCTTAG** |
| 5'-STM0847-delFRT_fw | TTATAGTAAAAGTTGTGTGCCACCGTAATGGACCCTAAAA**GTGTAGGCTGGAGCTGCTTC** |
| 3'-STM0847-delFRT_rv | GCGGCTATCCAGTTCATCGCTGTGCGGCACCTGCGGCTCC**CATATGAATATCCTCCTTAG** |
| 5'-STM1268-delFRT_fw | TATTAAGATAACATTAAGTAATTATTACAGATGGGTATTA**GTGTAGGCTGGAGCTGCTTC** |
| 3'-STM1268-delFRT_rv | TGAGCGGGGGAGAGAAATTAGTGACCTATTAAAGTTAGAC**CATATGAATATCCTCCTTAG** |
| 5'-rygD-delFRT_fw | TCGCGGTTGACGCTGCTTTTTTTTCGACCTAAAGTAAAGG**GTGTAGGCTGGAGCTGCTTC** |
| 3'-rygD-delFRT_rvA | CACTCACCAACAATCGCGATGTCAGCAAGGGGCTGAAACG**CATATGAATATCCTCCTTAG** |
| 5'-STM0971-FKF_fw | CATCCTGTAAAAAAGCGGTACTGCGAGCGTAAATTTTGGA**GTGTAGGCTGGAGCTGCTTC** |
| 3'-STM0971-FKF_rv | ATCAGCCTGGGTTTAGAAATGACATCAGATTTACACCTGA**CATATGAATATCCTCCTTAG** |
| 5'-STM1267-FKF_fw | CTTGCCGCCTTTAAGCAACTCGAATTATTTTGGGTATATA**GTGTAGGCTGGAGCTGCTTC** |
| 3'-STM1267-FKF_rv | CAGGGAGATCGGCTTCAATTTACATCGAATATGGTGAAAT**CATATGAATATCCTCCTTAG** |
| 5'-STM1896-FKF_fw | GCTGGCGTCAGCTAATAGCCTCACGGAAACACGGGAAAGA**GTGTAGGCTGGAGCTGCTTC** |
| 3'-STM1896-FKF_rv | AATTAATGCTGTGCATTAAACTGTGATGGTAATCTGGCAA**CATATGAATATCCTCCTTAG** |
| 5'-STM3363-FKF_fw | ACGACTTGCCCCCGCCTGCCGGGGGCTTTTTTATGCTTTT**GTGTAGGCTGGAGCTGCTTC** |
| 3'-STM3363-FKF_rv | AGTAAAACAGACAATTGTCTGTCGGGGATGGTGGCTGGCT**CATATGAATATCCTCCTTAG** |
| 3'-sciA_FKF_rv | GGCTTAACCCACTGAGAGTAATGATATGGACATGTCCCTG**CATATGAATATCCTCCTTAG** |
| 5'-sciA_FKF_fw | GGAGAACCGCCAGACATGCGCATCATCACTCCAGGGCATC**GTGTAGGCTGGAGCTGCTTC** |
| 3'-STM0295_FKF_rv | CGGGAAAAGATAACCCCTCCGGCTTTTACACATTATTCAT**CATATGAATATCCTCCTTAG** |
| 5'-STM0295_FKF_fw | AGTAACGGATTTGCTTGCTGGAATTGTGTTAACGAATGCA**GTGTAGGCTGGAGCTGCTTC** |
| 3'-STM1575_FKF_rv | GATTTTTATTAAATTATTTGTAACTTTACGCAAAGAGTTC**CATATGAATATCCTCCTTAG** |
| 5'-STM1575_FKF_fw | GCGCATTGGCTAATGTGGGGAAAATATGAGTTACCTTAAC**GTGTAGGCTGGAGCTGCTTC** |
| 3'-STM1630_FKF_rv | ATATTTCTTAAGTGAGTTAACTATAATGAATGATGATTCA**CATATGAATATCCTCCTTAG** |
| 5'-STM1630_FKF_fw | CACGCTCTTGCCAACCAGCATTTTATTAAAGCCTGACTTT**GTGTAGGCTGGAGCTGCTTC** |
| 3'-vrgS_FKF_rv | ATGAGAACTCCAGGTTTAATTCAGATCACTCAGAGGTGAG**CATATGAATATCCTCCTTAG** |
| 5'-vrgS_FKF_fw | ATCCGGTATTAAAGGAGTCACTACCATGAGTTTTGTATCC**GTGTAGGCTGGAGCTGCTTC** |
| 3'-YJCC_FKF_RV | TCGATCGCACTCCCAGCGATTACCGTCACCGCCTGTAGGG**CATATGAATATCCTCCTTAG** |
| 5'-yjcC_FKF_fw | AATAATGCAGGGAAATAGGCTGAAAATGAGTCAAAGCACA**GTGTAGGCTGGAGCTGCTTC** |
| gyrB_qPCR_new-fw | ACGCTCTGTCGCAAAAACTG |
| gyrB_qPCR_new-rv | ACCATCGTGCCGGTTTTATC |
| gmk_qPCR_new-fw | TTTTGCCGCCGTCAAAGATC |
| gmk_qPCR_new-rv | ATGGCTCATTTCTGCAACCG |
| rpoD_qPCR_new-fw | ACACCATCAAAGCGAAAGGC |
| rpoD_qPCR_new-rv | TCATCACGCGCATACTGTTG |
| 5'-csgB_qPCR_fw | ATTGAGCAAACGGGCAATGC |
| 3'-csgB_qPCR_rv | ACTGCTGTTTTCTGCGTACC |
| 5'-csgA_qPCR_fw | AGCATTCGCAGCAATCGTAG |
| 3'-csgA_qPCR_rv | AATGCTCAACGTGGAATCCG |
| 5'-fimZ_qPCR_fw | GGCGCAAACGGATTTGTAAG |
| 3'-fimZ_qPCR_rv | TTTGGGGGTACGGGTATTACTG |
| 5'-fimW_qPCR_fw | ATTTGCCACTACTGCACGAC |
| 3'-fimW_qPCR_rv | AAAGTAAAGCGGCCGTTTCG |
| 5'-fimH_qPCR_fw | TAACGTGTCGCAGCAAAAGC |
| 3'-fimH_qPCR_rv | AACATTGTCTGGCGAGGGATC |
| 5'-pefA_qPCR_fw | AGCGTGAACTCCAAAAACCC |
| 3'-pefA_qPCR_rv | TCAGCTTGGCTTTGAACTGC |
| 5'-siiC_qPCR_fw | TGTCTCAGGAGGTTGTGACAAC |
| 3'-siiC_qPCR_rv | TACACCGGAAGATACCAGCAAC |
| 5'-siiE_qPCR_fw | AGCGCTCAATGAAGCGTTTG |
| 3'-siiE_qPCR_rv | ATTTTGCTGGGAAGCATCGC |
| 5'-stdA_qPCR_fw | AATACCGCCAATGCAACCAC |
| 3'-stdA_qPCR_rv | ATCTGCTGTGCCACTGAAAG |
