# Supplementary material for: Characterization of Novel Factors Involved in Swimming and Swarming Motility in Salmonella enterica Serovar Typhimurium
Source: PLoS One. 2015 Aug 12;10(8):e0135351. doi: 10.1371/journal.pone.0135351 (PMC4534456; doi:10.1371/journal.pone.0135351)
Supplement: S3 Table — (DOCX) [file pone.0135351.s007.docx]

**Table S3:**

Doubling times and growth rates of single gene deletion mutants.

| **Mutation** | **Doubling time**  **(time [h] per generation)** | **Growth rate**  **(# of doublings per time [h])** |
| --- | --- | --- |
| WT | 1.55 | 0.447 |
| ΔSTM0266::FRT | 1.46 | 0.4733 |
| Δ*ydiV*::FKF | 1.77 | 0.3926 |
| ΔSTM0295::FRT | 1.46 | 0.4757 |
| Δ*rfaG*::FRT | 1.73 | 0.4005 |
| ΔSTM1575::FRT | 1.48 | 0.4695 |
| Δ*rygD*::FRT | 1.27 | 0.5439 |
| Δ*fliB* | 1.32 | 0.5261 |
| ΔSTM1630::FRT | 1.32 | 0.5255 |
| Δ*fljA*::FKF | 1.51 | 0.4579 |
| ΔSTM0971::FRT | 1.68 | 0.412 |
| Δ*yjcC*::FRT | 1.3 | 0.5331 |
| ΔSTM1267::FRT | 1.48 | 0.4676 |
| ΔSTM0289::FRT | 1.37 | 0.5063 |
| Δ*fimZ*::FKF | 1.39 | 0.4988 |
| ΔSTM1896::FRT | 1.29 | 0.5359 |
| ΔSTM0847::FRT | 1.4 | 0.4959 |
| Δ*sipA*::FKF | 1.46 | 0.4764 |
| ΔSTM3363::FRT | 1.36 | 0.5096 |
| ΔSTM1131::FRT | 1.36 | 0.5102 |
| Δ*sptP*::FKF | 1.6 | 0.4332 |
| ΔSTM1268::FRT | 1.16 | 0.5952 |
| Δ*flgE* | 1.32 | 0.5259 |
| ΔSTM3696::FRT | 1.48 | 0.4675 |
| Δ*fliH* | 1.63 | 0.4252 |
