# Supplementary material for: Characterization of Novel Factors Involved in Swimming and Swarming Motility in Salmonella enterica Serovar Typhimurium
Source: PLoS One. 2015 Aug 12;10(8):e0135351. doi: 10.1371/journal.pone.0135351 (PMC4534456; doi:10.1371/journal.pone.0135351)
Supplement: S4 Table — Significantly increased or decreased motility phenotypes are shown in bold. (DOCX) [file pone.0135351.s008.docx]

**Table S4:**

Motility phenotypes of single gene deletion mutants. Significantly increased or decreased motility phenotypes are shown in bold.

| **Strain number** | **Relevant genotype** | **Relative swimming motility** | **Relative swarming motility** |
| --- | --- | --- | --- |
| TH6622 | ATCC14028s wildtype | 1 ± 0.12; n = 20 | 1 ± 0.32; n = 34 |
| EM2383 | Δ*fimZ*::FKF | 0.99 ± 0.19; n = 10 | 0.86 ± 0.25; n = 23 |
| EM2590 | Δ*flgE* | **0.09** ± 0.04; n = 6 | **0.09** ± 0.01; n = 6 |
| EM880 | Δ*fliB* | 1.00 ± 0.12; n = 6 | 1.24 ± 0.45; n = 9 |
| EM824 | Δ*fliF* | **0.1** ± 0.02; n = 10 | **0.04** ± 0; n = 5 |
| EM2591 | Δ*fliH* | **0.09** ± 0.06; n = 6 | **0.1** ± 0.02; n = 6 |
| EM2381 | Δ*fljA*::FKF | 0.95 ± 0.1; n = 10 | 1.1 ± 0.21; n = 23 |
| EM2605 | Δ*rfaG*::FRT | **0.27** ± 0.06; n = 10 | **0.03** ± 0; n = 13 |
| EM1691 | Δ*rygD*::FRT | 0.98 ± 0.05; n = 20 | 1.01 ± 0.33; n = 20 |
| EM2384 | Δ*sipA*::FKF | 0.98 ± 0.09; n = 10 | 1.02 ± 0.35; n = 13 |
| EM2385 | Δ*sptP*::FKF | 0.97 ± 0.09; n = 10 | 1.06 ± 0.33; n = 12 |
| EM1507 | ΔSTM0266::FRT | 1 ± 0.09; n = 15 | 1.08 ± 0.19; n = 8 |
| EM1512 | ΔSTM0289::FRT | 0.95 ± 0.12; n = 15 | 1.03 ± 0.18; n = 9 |
| EM1508 | ΔSTM0295::FRT | 1.03 ± 0.07; n = 15 | 1.04 ± 0.19; n = 8 |
| EM1686 | ΔSTM0847::FRT | 1 ± 0.06; n = 20 | 1.11 ± 0.3; n = 17 |
| EM1480 | ΔSTM0971::FRT | 1.02 ± 0.15; n = 9 | 0.98 ± 0.34; n = 4 |
| EM1688 | ΔSTM1131::FRT | 0.97 ± 0.06; n = 20 | 0.93 ± 0.19; n = 5 |
| EM1481 | ΔSTM1267::FRT | 0.99 ± 0.04; n = 9 | **1.31** ± 0.23; n = 14 |
| EM1689 | ΔSTM1268::FRT | 1.01 ± 0.09; n = 20 | 1.06 ± 0.22; n = 18 |
| EM1509 | ΔSTM1575::FRT | 0.99 ± 0.09; n = 15 | 0.93 ± 0.24; n = 9 |
| EM1510 | ΔSTM1630::FRT | 1 ± 0.13; n = 29 | 0.93 ± 0.26; n = 10 |
| EM1482 | ΔSTM1896::FRT | 1.05 ± 0.09; n = 9 | 1.09 ± 0.42; n = 4 |
| EM1484 | ΔSTM3363::FRT | 1.02 ± 0.13; n = 9 | **1.36** ± 0.21; n = 14 |
| EM1690 | ΔSTM3696::FRT | 1.01 ± 0.09; n = 20 | 1.09 ± 0.26; n = 18 |
| EM2382 | Δ*ydiV*::FKF | **1.57** ± 0.14; n = 10 | **0.60** ± 0.15; n = 13 |
| EM1511 | Δ*yjcC*::FRT | **0.81** ± 0.12; n = 15 | 1.04 ± 0.2; n = 10 |
